# Lithotomy versus jack-knife position on haemodynamic parameters assessed by impedance cardiography during anorectal surgery under low dose spinal anaesthesia: a randomized controlled trial

**DOI:** 10.1186/s12871-015-0055-3

**Published:** 2015-05-06

**Authors:** Jurgita Borodiciene, Jurate Gudaityte, Andrius Macas

**Affiliations:** Department of Anaesthesiology, Lithuanian University of Health Sciences, Eiveniu str. 2, LT-50009 Kaunas, Lithuania

**Keywords:** Spinal anaesthesia, Haemodynamic parameters, Cardiac output, Impedance cardiography, Prone position

## Abstract

**Background:**

Although the prone position providing better exposure for anorectal surgery is required it can cause a reduction of cardiac output and cardiac index. The goal was to compare haemodynamic changes assessed by impedance cardiography during anorectal surgery under low-dose spinal anaesthesia in lithotomy and jack-knife position.

**Methods:**

The prospective randomized controlled study included 104, ASA I-II adult patients admitted for elective minor anorectal surgery, assigned to be performed in lithotomy (groupL, n = 52) or jack-knife position (groupJ, n = 52). After arrival to operating room the standard monitoring, impedance cardiography device was connected to the patient, and the following variables were recorded: cardiac output, cardiac index, systemic vascular resistance, stroke index at times of arrival to operating room, placement for, start and end of surgery and placement to bed. Spinal block was made in the sitting position with 4 mg of 0.5% hyperbaric bupivacaine and 10 μg of Fentanyl injected over 2 min. Comparison was based on haemodynamic changes between and inside groups over time. Student’s t, chi square tests were used for statistical analysis with p < 0.05 regarded as statistically significant.

**Results:**

The reduction of cardiac output was statistically significant after placement of the patient into the prone position: from baseline 7.4+/−1.6 to 4.9+/−1.2 after placement for and 4.7+/−1.2 at the start and end of surgery (mean +/−SD l/min). The difference of cardiac output between groups was 2.0 l/min after positioning for and the start of surgery and 1.5 l/min at the end of surgery (p < 0.05). Mean cardiac index reduced from baseline 3.9+/−0.8 to 2.6+/−0.7 and 2.4+/−0.6 (mean+/−SD l/min/m^2^) in groupJ and between groups: by 1.0 l/min/m^2^ after placement for, 1.1 at the start and 0.8 at the end of surgery (p < 0.05). Systemic vascular resistance increased from baseline 1080+/−338 to 1483+/−479 after placement for, 1523+/−481 at the start and 1525+/−545 at the end of surgery in groupJ (mean+/−SD dynes/sec/cm^−5^, p < 0.05).

**Conclusions:**

According to impedance cardiography, jack-knife position after low-dose spinal anaesthesia produces transitory, but statistically significant reduction of cardiac output and cardiac index with increase of systemic vascular resistance, compared to insignificant changes in lithotomy position.

**Trial registration:**

Clinical Trials NCT02115178.

## Background

According to the Helsinki declaration on patient safety in anaesthesiology and the requirements for ambulatory surgery, we must achieve the 100% safety [1]. To reach this, comprehensive knowledge on anaesthetic methods as well as on physiological changes of the preoperative period is essential. The patient position required for surgery affects the cardiac function and haemodynamic parameters [2]. The placement into a prone position for surgery can cause an immediate reduction of cardiac output (CO), cardiac index (CI) and other haemodynamic parameters [2-4]. A number of patient’s different positions can be used for anorectal surgery. Minor anorectal procedures, like haemorrhoidectomy, excision of the anorectal fistula, etc. are usually performed in the prone (jack-knife) or lithotomy position. As the prone position is required to provide a better surgical access to some anorectal operations, predictable changes in physiology as well as a number of complications may occur: injuries of central and peripheral nervous system, injuries due to arterial and venous occlusion, ophthalmic injuries and pressure injuries [2,4]. The placement into a prone position can cause significant physiological cardiovascular and respiratory effects: a decrease in the arterial blood pressure, changes in the heart rate (HR), CI, CO and other parameters, particularly in the cases of the advanced age, cardiovascular diseases and other co-morbidities. According to Schmittner et al., spinal anaesthesia with a low dose of hyperbaric local anaesthetic is superior to general anaesthesia for colorectal procedures in terms of postoperative analgesic consumption, recovery time, the rate of postoperative complications and patient satisfaction [4]. A standardized selective spinal anaesthesia with a low dose of hyperbaric bupivacaine for anorectal surgery produces a sufficient level of sensory and motor block and the quality of anaesthesia with a shorter duration and faster recovery than the general anaesthesia or the conventional spinal anaesthesia [5]. A number of studies investigating haemodynamic changes in the prone position are limited and even more limited concerning lithotomy position [6-12].

The purpose of this prospective randomized clinical study was to investigate and compare the changes in CO, CI, systemic vascular resistance (SVR) and stroke index (SI) assessed by impedance cardiography (ICG) during anorectal surgery under a low-dose spinal anaesthesia in lithotomy and jack-knife position.

## Methods

### Patient population

After the approval from Local Ethics Committee (Kaunas Regional Biomedical Research Ethics Committee, ref. n. BE-2-15) and international registration (http://www.clinicaltrials.gov, registration number NCT02115178), followed by a written informed consent, 104 ASA physical status 1 – 2 adult patients admitted for elective anorectal surgery, were recruited into this prospective, observational, single–centre randomized controlled study. Exclusion criteria were as follows:

- contraindications for regional anaesthesia,
- cardiac arrhythmias,
- history of severe chest, heart and lung diseases (e.g. chest deformities, pulmonary edema, pleural and pericardial effusions, intracardiac shunts, failure of aortic valve, foreign bodies (e.g. chest tube)),
- pregnancy,
- gross obesity (body mass index (BMI) > 35),
- chronic treatment with psychotropic and analgesic drugs,
- patients whose movements on the operating room (OR) table, including shivering, could not be controlled,
- height < 120 or > 230 centimeters and weight < 30 or > 150 kilograms.

### Anaesthesia and monitoring

After arrival to the OR, standard monitoring: ECG, pulseoximetry and non-invasive blood pressure, were started before anaesthesia. According to the manufacturer’s manual, two pairs of dual sensors (i.e. eight electrodes in total) were placed on both sides of the patient’s neck and two pairs on both sides of the chest and connected to the ICG device (Niccomo™, Medis. Medizinische Messtechnik GmbH, Ilmenau, Germany). The skin was prepared in the way similar to the electrocardiography examination. Current intensity and frequency were 1.5 mA and 86 kHZ, respectively. The SV values were calculated adjusting them to the patient‘s gender, height and body weight and excluding distortions and artefacts in the ICG signal. The quality of the signal with the value of quality indicator over 80% was regarded as sufficient for further calculations [13]. The ICG was set to express CO by taking the mean value of 16 heart beats. The SV was calculated beat by beat and averaged over the periods of 30 sec. The configuration of the electrodes was not changed and the head was not turned to any side and was kept in a straight line with the body.

A dedicated researcher was responsible for monitoring, measurements and data registration. The peripheral intravenous access was established and the Ringer’s acetate (FreseniusKabi) infusion was initiated. Intravenous fluids were restricted to 5–7 ml/kg/h. preoperatively. No bolus of intravenous fluids to increase preload was administered before anaesthesia.

All patients were premedicated with oral 5 mg diazepam and 100 mg diclofenac.

**Figure 1:**
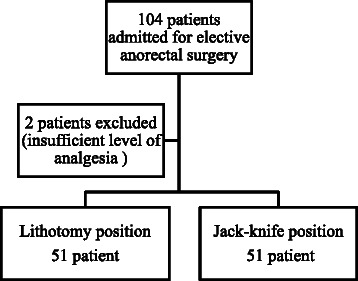
Flow diagram.

Spinal anaesthesia was performed in the sitting position with a 27G Tamanho spinal needle (B. Braun, Melsungen, Germany) by using a median approach. After aspiration, 4 mg (0.8 ml) of 0.5% hyperbaric bupivacaine (Marcaine Spinal Heavy; Astra Zeneca, Lund, Sweden) and 10 μg (0.2 ml) of fentanyl were injected over two minutes. After sitting for 10 min, the level of sensory block was tested with the alcohol swab and the level of a motor block was assessed with the Bromage score. The patients were blindly randomized in which position they were placed by a sealed envelope method before arrival to the OR. The patients were placed into one of the two positions: lithotomy (group L) or a prone jack-knife (group J) and the surgery was started. Three anaesthesiologists performed a spinal block and positioning of the patients. The patients were sedated with 1–5 mg of intravenous midazolam. In the cases of the failed block, general anaesthesia was induced, and these patients were excluded from the study (Figure 1). A decrease in systolic BP below 90 mmHg was treated with 5 mg of intravenous (IV) ephedrine and HR < 45 BPM with 0.5 mg of atropine.

The following variables were recorded: systolic and diastolic arterial blood pressure, MAP, HR, CO, CI, SI, calculated SVR. The parameters were recorded at eight time points: at the time of arrival to OR (baseline), the placement in the sitting position, immediately after and 10 min after the dural puncture, the placement for surgery, start and end of the surgery and the placement to bed. The comparison was based on haemodynamic changes between and inside the groups over time.

Anaesthesia and the surgical technique variables: the level of puncture (L2 - L3, L3 - L4, or L4 - L5), the level of sensory (dermatomes) and the motor block (Bromage score 0–3) after 10 min, the type and duration of surgery and anaesthesia in minutes, were recorded.

The balanced postoperative analgesia was provided with non-steroidal anti-inflammatory drugs – 100 mg of oral diclofenac every 12 hours and 50 mg of intramuscular pethidine for rescue analgesia. The patients after surgery were treated in the surgical ward.

### Statistical analysis

The statistical analysis was performed by using IBM SPSS statistical software (SPSS v.21.0 for Windows). To determine the required sample size with a power of 80%, a cardiac index was considered as the primary efficacy variable. It is believed that the difference of 0.5 l/min/m^2^ in the cardiac index between the supine and jack-knife position is of clinical importance. Assuming that the standard deviation of 0.7 l/min/m^2^ is obtained from the previous studies [7,8], a sample size of 50 patients for detection of a the difference of 0.5 l/min/m^2^ in a cardiac index with the power of 80% and at α = 0.05 when performing a two – tailed test was required in each group. To compensate for possible losses, 52 patients were recruited in each group. The normality (Kolmogorov – Smirnov) test was applied to all data. The students’ t - test, chi square test and pairwise comparisons were used to compare changes in haemodynamic parameters. The repeated – measures analysis of variance (ANOVA) was performed to compare serial changes in haemodynamic parameters between the two groups to determine the time at which a significant difference was found.

## Results

The randomized controlled study included 104 patients admitted for elective minor anorectal surgery. Two patients were excluded due to insufficient level of analgesia (Figure 1).

The patient demographic data are summarized in Table 1. The study population consisted of 25 men and 26 women in group J versus 24 and 27 in group L, the mean age 46.2 ± 14 years in group J versus 49.2 ± 13.4 years in group L (p > 0.05). The groups were comparable with respect to the mean body weight, height, age, and the level of sensory and motor block.

**Table 1:** **Patient demographics**

Variable	Jack – knife (prone) position	Lithotomy position
	n = 51	n = 51
Age (yrs)	46.2 ± 14	49.2 ± 13.4
	(20 – 74)	(25 – 79)
Weight (kg)	80.9 ± 14.3	81.1 ± 14.6
	(54 – 110)	(46 – 115)
Height (cm)	173.1 ± 9.1	173.9 ± 10.1
	(154 – 187)	(142 – 198)
Sensory level after 10 min, dermatomes	L2 – 1	L2 – 1
	L3 – 42	L3 – 40
	L5 – 5	L5 – 8
	S1 – 3	S1 – 2
Sex (F/M)	26/25	27/24
ASA I/II	19/33	21/31

ASA - American Society of Anesthesiologists (physical status classification system).

Values are mean ± SD (range) or ratio.

The type, duration of surgery and anaesthesia are presented in Table 2. The groups were comparable regarding these variables. Haemorrhoidectomy was performed for 43.1% of patients. The difference of duration of anaesthesia and surgery was 18 min in group L and 14 min in group J.

**Table 2:** **Duration of surgery and anaesthesia, type of surgery**

Variable	Jack – knife (prone) position	Lithotomy position
**Type of surgery:**		
Haemorhoidectomy	24	20
Excision of anorectal fistula	9	11
Discision of anorectal fistula	8	10
Other	10	11
**Duration of surgery (min)**	25 ± 9.3	26 ± 15
**Duration of anaesthesia (min)**	39 ± 9.9	44 ± 13

Values are mean ± SD or number of cases.

**Figure 2:**
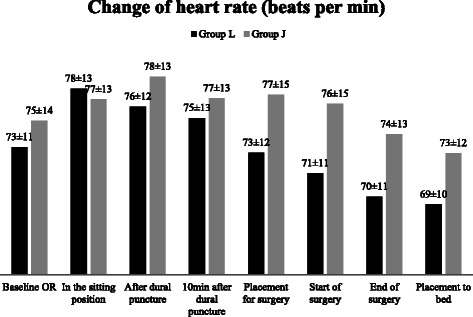
Changes of heart rate (HR). Data are presented as mean ± SD. No significant difference between or inside groups.

**Figure 3:**
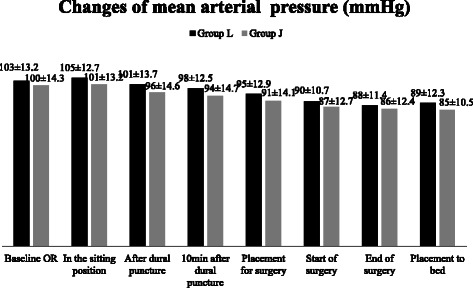
Changes of mean arterial pressure (MAP). Data are presented as mean ± SD, no significant difference between or inside groups.

**Figure 4:**
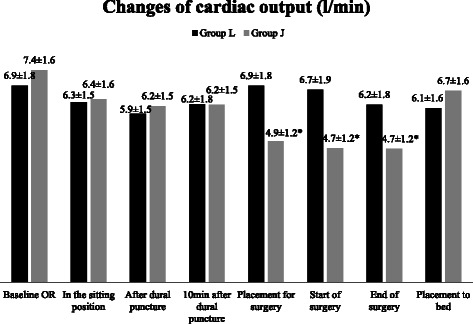
Changes of cardiac output (CO). Data are presented as mean ± SD, *p < 0.05 considered as statistically significant group J vs group L and baseline vs jack - knife position.

**Figure 5:**
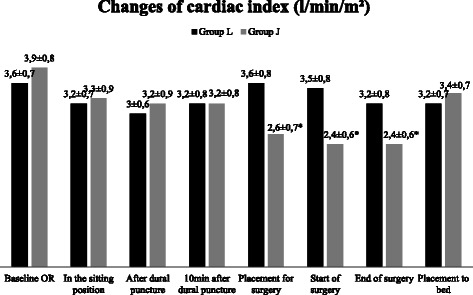
Changes of cardiac index (CI). Data are presented as mean ± SD, *p < 0.05 considered as statistically significant group J vs group L and baseline vs jack - knife position.

**Figure 6:**
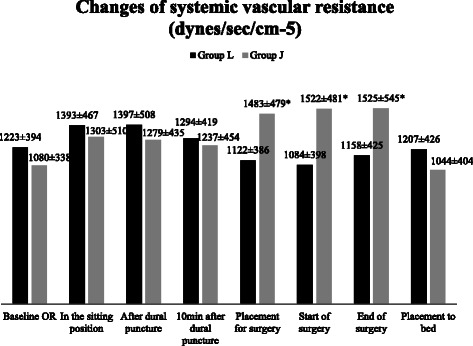
Changes of systemic vascular resistance (SVR). Data are presented as mean ± SD, *p < 0.05 considered as statistically significant group J vs group L and baseline vs jack - knife position.

**Figure 7:**
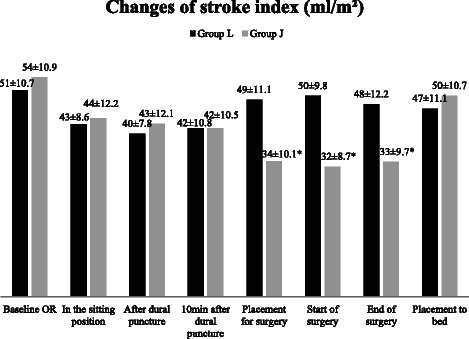
Changes of stroke index (SI). Data are presented as mean ± SD, *p < 0.05 considered as statistically significant group J vs group L and baseline vs jack - knife position.

The changes in haemodynamic variables are presented in Figures 2, 3, 4, 5, 6 and 7. The changes of MAP and HR were comparable between the groups or inside the groups (Figures 2 and 3).

The CO decreased statistically significantly after the placement into the jack-knife position: by 2.5 l/min (from the baseline (mean (l/min) ± SD) 7.4 ± 1.6 to 4.9 ± 1.2 (p < 0.05)) immediately after the patient’s positioning and by 2.7 l/min (p < 0.05) at the start and at the end of surgery, i.e. the reduction of CO was by 33.8% from the baseline after the placement into the jack-knife position. On the contrary, CO remained stable or decreased slightly in the lithotomy group (the reduction of CO was by 11.1% from the baseline). In addition, the difference between the groups in CO was by 2.0 l/min at the time of placement for the surgery followed by 1.5 l/min at the end of surgery (p < 0.05) (Figure 4).

The changes in CI were recorded as follows (Figure 5): reduction by 1.3 l/min/m^2^ (from baseline (mean (l/min/m^2^) ± SD) 3.9 ± 0.8 to 2.6 ± 0.7 (p < 0.05)) after the placement in the jack-knife position and 1.5 l/min/m^2^ at the start and at the end of the surgery. The reduction of CI was by 33.3% from the baseline in the jack-knife group versus by 17.9% in the lithotomy group. The difference between the groups was by 1.0 l/min/m^2^ after positioning for, by 1.1 l/min/m^2^ at the start and 0.8 l/min/m^2^ at the end of surgery (p < 0.05).

There was also a significant change in SVR (Figure 6). SVR (mean ± SD) increased from 1080 ± 338 at baseline to 1483 ± 479 dynes/sec/cm^**−5**^ (p < 0.05) after the patient’s placement into the jack-knife position, and the increase remained stable (1523 ± 481 and 1525 ± 545 dynes/sec/cm^**−**5^) at the start and at the end of the surgery (p < 0.05). The difference between the groups according to the time was by 24.3% after the placement in the jack-knife position, 28.8% at the start and 24.1% at the end of surgery (p < 0.05).

SI was reduced significantly after the placement into the jack-knife position: by 20.6 ml/m^2^ (from 54.2 ± 10.9 at the baseline to 33.6 ± 10.2 ml/m^2^ (mean ± SD, p < 0.05)) immediately after the patient’s positioning and by 22 ml/m^2^ at the start and by 20.6 ml/m^2^ at the end of the surgery. The reduction of SI was by 38% from the baseline after the positioning into the jack-knife group. On the contrary, SI remained stable or decreased slightly in the lithotomy group (SI was reduced by 4.7% from the baseline). In addition, the difference between groups in SI was by 15.1 ml/m^2^ at the time of placement for the surgery followed by 14 ml/m^2^ at the end of the surgery (p < 0.05) (Figure 7).

## Discussion

The main focus of this prospective randomized clinical study was determination of the position dependent haemodynamic changes during the minor anorectal surgery under a low-dose spinal anaesthesia assessed by ICG. In our study, the reduction of CI by 33.3% (1.3 l/min/m^2^ (from the baseline 3.9 ± 0.8 to 2.6 ± 0.7)) and CO by 33.8% (2.5 l/min (from the baseline 7.4 ± 1.6 to 4.9 ± 1.2)) and the increase of SVR from the baseline immediately after the patient’s positioning was determined in the jack-knife position. CO, CI, SVR, SI remained stable or changed slightly in the lithotomy position. The MAP and HR remained stable in both positions.

Assuming that a low dose spinal anaesthesia has negligible haemodynamic effects, we initiated this study with the aim of evaluation of haemodynamic changes and effects of the patient’s positioning on haemodynamic parameters assessed by the transthoracic electric bioimpedance (TEB). The second goal to start this study was that we could find no currently available studies of haemodynamic effects assessed by the non-invasive method of CO monitoring during a minor anorectal surgery under the selective spinal anaesthesia in two different positions: prone or lithotomy position.

For anorectal surgery, the jack-knife position with the head down tilt of 15–20 degrees for easier surgical access is frequently used. The majority of surgeons in the Hospital of Lithuanian University of Health Sciences, Kaunas Clinics prefer to use the jack-knife position than the lithotomy position for the anorectal surgery.

The well – known haemodynamic effects of the conventional dose spinal anaesthesia are arterial hypotension and bradycardia [14,15]. They are caused by multiple mechanisms: 1) extensive neuraxial blockade including the sympathetic chain, 2) redistribution of intravascular volume due not only to vasodilation, but also to the patient’s positioning, and 3) paradoxical vasovagal reactions [15]. The decrease in pre-load that may accompany spinal anaesthesia or the patient’s position, may initiate the following three reflexes that can eventually lead to a sudden onset of cardiovascular collapse and syncope. The first reflex involves direct stretching of the pacemaker cells in the sinoatrial node. The decrease in venous return produces less stretch and a lower heart rate. The second reflex involves baroreceptors located within the walls of the right atrium and the vena-cava–atrial junction. The stimulation of these receptors by an increase in venous return sends signals to the vasomotor centre. The decreases in venous return induce a decrease in the heart rate. The third (Bezold–Jarisch) reflex is mediated by cardiac baroreceptors located in the inferoposterior wall of the left ventricle. This reflex provokes a decrease of central blood volume with decreases of ventricular volume and an increase of ventricular contractility. This leads to a combination of the increase in vagal efferent activity from the vasomotor centre leading to varying degrees of bradycardia and a decrease in the efferent sympathetic output to the primary sympathetic neurons in the thoracolumbar spinal cord leading to marked vasodilation [15].

To provide a safe care, the anaesthesiologist must maintain tissue perfusion and haemodynamic stability by ensuring optimal preoperative fluid balance. This can be achieved by monitoring of cardiovascular function by using the invasive CO monitoring, such as pulmonary artery catheterization – thermodilution method, less invasive such as oesophageal Doppler monitor or non-invasive cardiac output monitoring achieved by TEB [16-18]. CO is a primary determinant of global oxygen transport from the heart to the body. So, monitoring of CO, CI and other haemodynamic parameters may have a role in monitoring circulation and forming management decisions. The patients in our study were awake, under selective spinal anaesthesia with low dose hyperbaric bupivacaine, admitted for elective minor anorectal surgery and relatively healthy (ASA class 1–2). Therefore, the non-invasive method of monitoring a cardiovascular function – TEB was chosen for this study due to a number of factors: safety, convenience, adaptability, cost, type of surgery and anaesthesia and the possibility of using it for these patients [16,17].

Sudden haemodynamic reactions occur despite that the selective low dose spinal anaesthesia affecting restricted dermatomes was used [5,19]. Gudaityte et al. [5] concluded that the selective spinal anaesthesia with a dose of 4 mg of hyperbaric bupivacaine produces an adequate level of sensory and motor block for anorectal surgery. Despite the fact that MAP was stable, acute bradycardia was recorded in 6 cases before a dural puncture in the sitting position and in 2 cases immediately after the injection of local anaesthetic. In our study, the patients were relatively healthy, and haemodynamic changes were not clinically significant despite the reduction of CO by 33.8%, and no further rescue treatment was needed. We failed to measure ICG during the cases of sudden bradycardia (three cases, all prior to dural puncture). The drawback of ICG is that it requires time to get a signal of sufficient quality. We could not proceed with prolonged investigation due to ethical issues, or use controlled measurements by thermodilution with a pulmonary catheter after the assessment of a potential risk/benefit ratio.

The prone or the jack-knife position itself is associated with physiological cardiovascular and respiratory changes, and the reduction of CI and CO is most frequent [2]. The researchers suggest that the anaesthetic technique could affect haemodynamic variables in the prone position [5,7,20]. Our findings are comparable to other studies [6,9,12,21]. Several studies revealed the decrease of CO by 18.5% - 24.4% during a lumbar surgery in the prone position under general anaesthesia [6,10]. The authors concluded that the reduction was mainly due to a reduction in a stroke volume index. The transoesophageal dopller (TEE) was used to determine these findings [6]. Others studies reported that changing the patient’s position to prone significant decreases CI by 17.2% and increases the total SVR, but there is little change in other haemodynamic variables [8,9]. The researchers of this study used the invasive method of cardiovascular function monitoring, i.e. thermodilution method. Ozkose et al. compared total intravenous anaesthesia (TIVA) with inhalation anaesthesia by measuring MAP and HR in patients undergoing spinal surgery. HR and MAP decreased significantly after induction of anaesthesia in the TIVA group compared to other groups when the patients were placed in the prone position [20]. Sudheer et al. used a non-invasive cardiac output NICO™ system - partial rebreathing of the carbon dioxide technique to measure CI. They compared the impact of two different methods of anaesthesia (inhalation and TIVA) to CI and SVR during the lumbar spinal surgery and found the significant reduction of CI by 25.9% in TIVA group and by 12.9% in inhalation group and the increase in SVR on turning the patients prone [7].

Wadsworth and colleagues [22] used TEB to measure CO and HR. CI and the total vascular resistance index were derived from these data. No significant changes in HR or MAP occurred when the volunteers were positioned from the supine position to any of the four prone positions or when returned to the supine position again. CI decreased significantly on turning from the supine to the knee-chest position (20%) and onto the two props with a support (17%), but not onto the evacuatable mattress (11%) or two pillows (3%) [22]. A reduction in CI is due to a decrease in venous circulation from venostasis in the lower extremities and an increase in thoracic pressure. However, the change in blood pressure is not common due to the result of the increase in total systemic vascular resistance caused by the activation of the sympathetic nervous system following a decrease in CO [22]. Toytota and Amaki revealed controversial results [12]. They demonstrated that the prone position did not cause a reduction in CI and changes in the stroke volume index, however, caused a reduction of the left ventricular volume, systolic pulmonary venous flow velocity and pulmonary venous velocity time integral via TEE [12].

The studies investigating haemodynamic effects of the lithotomy position are limited. Miyabe et al. [11] concluded that the lithotomy position after spinal anaesthesia reduces a decrease in blood pressure and has no effect on the analgesic level [11]. They did not measure CO, CI or SVR. The results of our study revealed that MAP, HR, CO, CI, SVR, SI measured by ICG remained stable or changed slightly in the lithotomy position. The return of the pooled venous blood from the lower extremities (500–1000 ml) to the heart increased the afterload due to the elevation of the legs, assuming that baroreflex does not occur in the lithotomy position, explains the consistent result of lithotomy position [11].

There are many limitations to the validity of the calculation of cardiac output by ICG. These limitations can be divided into two main categories: 1) the difficulty of acquiring the signal because of spontaneous movements of the patient, interferences with electrocautery in OR, disorders of heart rhythm, or mechanical ventilation; the newest monitors, such as the Niccomo, include a quality indicator signal to eliminate real-time misinformation; and 2) physiologic and pathophysiologic situations in which the physical modelling of the system is no longer valid, in particular, during changes in the baseline thoracic impedance (pregnancy, obesity, gas or fluid pleural effusion, chronic congestive heart failure with pulmonary oedema), or when there is a severe aortic valve disease or modified mechanical properties of the arterial tree. These limitations must be kept in mind when using TEB at the bedside [13]. The jack-knife or lithotomy position can also affect the lung compliance and thoracic volume which may have impact to the accuracy of TEB measurements [2]. We avoided such conditions, as surgery in the chest, abdominal opening and closure, a vasodilation/vasoconstriction status, a lung fluid balance, an acute lung injury which can lead to the bias of ICG compared with thermodilution in the present study. The limitation of our study is that the single method of haemodynamic monitoring was used. We did not use a pulmonary artery catheter (PAC) as a controlled method for CO measurement. Monitoring with PAC is associated with complications and can hardly be justified if the risk for the patient and the cost of the device are taken into account. According to the risk/benefit ratio, the method of haemodynamic monitoring to this relatively healthy patient population should be non-invasive, accurate and should give reproducible results. The studies of ICG reported different results and are difficult to compare with each other because different ICG devices were used with different software and the subjects’ characteristics varied from healthy volunteers to the patients with heart failure, surgical procedures and medical treatment. In a meta - analysis, Peyton and Chong reported that the correlation between TEB and the pulmonary artery catheterization thermodilution method which is the “gold standard” for CO measurement is very good (r = 0.79). However, they demonstrated that the percentage error of ICG versus thermodilution is 42.9% with a significant precision error [16]. The relative changes of CO, CI, and SI may be accurate, but the absolute values may be different versus thermodilution method. According to the fact that ICG is a non-invasive, continuous, operator – independent and a cost effective tool for CO monitoring, we regarded this method as most acceptable for our study.

The present study was focused on the assessment of ICG changes in a relatively healthy, ASA 1–2 population with a normal compensatory reserve who could well tolerate haemodynamic shifts caused by the jack-knife position. We hypothesize that the haemodynamic changes after the placement into the jack-knife or prone position could be even more dramatic in the cases of a limited cardiovascular reserve, i.e. for patients with cardiovascular compromise.

## Conclusions

In conclusion, according to ICG, the placement of ASA class 1–2 patients into the jack-knife position for minor anorectal surgery under a low-dose spinal anaesthesia produces transitory, but statistically significant reduction of CO, CI and SI with the increase of SVR to maintain the MAP, compared to insignificant changes in the lithotomy position.

## Abbreviations

ICG: Impedance cardiography
CO: Cardiac output
CI: Cardiac index
SVR: Systemic vascular resistance
SI: Stroke index
ACI: Acceleration index
OR: Operating room
HR: Heart rate
BMI: Body mass index
bpm: Beats per minute
MAP: Mean arterial blood pressure
Group J: Jack-knife group
Group L: Lithotomy group
TEE: Transoesophageal dopller
TIVA: Total intravenous anaesthesia
IV: Intravenous
TEB: Transthoracic electric bioimpedance
